# Early Responses of *Brassica oleracea* Roots to Zinc Supply Under Sufficient and Sub-Optimal Phosphorus Supply

**DOI:** 10.3389/fpls.2019.01645

**Published:** 2020-01-09

**Authors:** Paula Pongrac, Sina Fischer, Jacqueline A. Thompson, Gladys Wright, Philip J. White

**Affiliations:** ^1^ Ecological Science Group, The James Hutton Institute, Dundee, United Kingdom; ^2^ Low and Medium Energy Physics, Jožef Stefan Institute, Ljubljana, Slovenia; ^3^ Future Food Beacon of Excellence and School of Biosciences, University of Nottingham, Loughborough, United Kingdom; ^4^ Distinguished Scientist Fellowship Program, King Saud University, Riyadh, Saudi Arabia

**Keywords:** mineral nutrition, kale, broccoli, element interaction, RNAseq, gene ontology

## Abstract

Shoot zinc (Zn) concentration in *Brassica oleracea* is affected by soil Zn and phosphorus (P) supply. Most problematic is the negative impact of P fertilizers on Zn concentrations in crops, which makes balancing yield and mineral quality challenging. To evaluate early molecular mechanisms involved in the accumulation of large shoot Zn concentrations regardless of the P supply, two *B. oleracea* accessions differing in root architecture and root exudates were grown hydroponically for two weeks with different combinations of P and Zn supply. Ionome profiling and deep RNA sequencing of roots revealed interactions of P and Zn *in planta*, without apparent phenotypic effects. In addition, increasing P supply did not reduce tissue Zn concentration. Substantial changes in gene expression in response to different P and/or Zn supplies in roots of both accessions ensured nutritionally sufficient P and Zn uptake. Numerous genes were differentially expressed after changing Zn or P supply and most of them were unique to only one accession, highlighting their different strategies in achieving nutrient sufficiency. Thus, different gene networks responded to the changing P and Zn supply in the two accessions. Additionally, enrichment analysis of gene ontology classes revealed that genes involved in lipid metabolism, response to starvation, and anion transport mechanisms were most responsive to differences in P and Zn supply in both accessions. The results agreed with previously studies demonstrating alterations in P and Zn transport and phospholipid metabolism in response to reduced P and Zn supply. It is anticipated that improved knowledge of genes responsive to P or Zn supply will help illuminate the roles in uptake and accumulation of P and Zn and might identify candidate genes for breeding high-yield-high-Zn brassicas.

## Introduction

Plants have an extraordinary ability to survive in a fluctuating environment. When facing scarcity in essential mineral elements (nutrients) plants employ a plethora of morphological, physiological and metabolic acclimatory mechanisms to enhance nutrient acquisition and/or their within-plant utilisation (White and Brown, 2010; White et al., 2013b). In the case of phosphorus (P) deficiency, enhanced phosphate (P_i_) acquisition is delivered by enhancing the mobilisation of P_i_ from soils, increasing the volume of soil explored by the root system to capture solubilized P_i_ and increasing the rate of P_i_ uptake across the root rhizodermis (Rose et al., 2013; Hunter et al., 2014; Wissuwa et al., 2016). Within-plant mechanisms reducing plant P-requirements include alterations in P translocation from younger to older tissues, utilisation of P-sparing metabolic pathways, reducing the abundance of nucleic acids, and recycling P through lipid remodelling (Veneklaas et al., 2012; Nakamura, 2013; Pant et al., 2015; Siebers et al., 2015; Tawaraya et al., 2018). In the case of zinc (Zn) deficiency, increased Zn uptake is achieved mainly by increasing Zn uptake capacity through a tightly regulated network of molecular interactions in which the coordinated expression of Zn transporters plays a major role in Zn acquisition from soil, in movement between organs and tissues and in intracellular sequestration of Zn (Assunção et al., 2010), although chemical modification of the rhizosphere to increase Zn phytoavailability and greater exploration of the root volume are also beneficial (White et al., 2013a). Adaptive traits involved in plant responses to P_i_ deficiency will also improve Zn acquisition (Rose et al., 2013). Thus, although the requirements for P and Zn differ by orders of magnitude, i.e. 2,000–5,000 µg P g^-1^ dry weight (DW) and 15–30 µg Zn g^-1^ DW in most crops (White and Brown, 2010) and their roles in the plants are contrasting, they are both poorly mobile in soil and as such, traits involved in the increased acquisition of one can improve the acquisition of the other.

Phosphorus is one of the key yield-limiting nutrients for crop production and responses to P_i_ deficiency and the optimisation of P-use-efficiency (PUE) have achieved significantly more attention than has improving Zn-use efficiency (ZnUE). This is unfortunate, since Zn deficiency in soils is a global problem and improving Zn nutrition of crops and increasing Zn concentration in seeds can have several beneficial effects including higher yields, better seed viability and seedling vigour, smaller seeding rate and improved tolerance to abiotic and biotic stress (Broadley et al., 2007; Cakmak, 2008; White and Veneklaas, 2012). In addition, Zn malnutrition (i.e. insufficient intake of Zn in animals and humans) remains a significant health problem, particularly in children (White and Broadley, 2009; Bouis and Saltzman, 2017; Cakmak and Kutman, 2018).

Recent findings suggest that there is a molecular coordination between P and Zn homeostasis (Huang et al., 2000; Bouain et al., 2014; Khan et al., 2014; Briat et al., 2015; Kisko et al., 2018) which may help interpret observations of P-Zn interactions in different plants. Three different types of P-Zn interactions have been reported: (1) P-induced Zn deficiency (Stukenholtz et al., 1966; Cakmak and Marschner, 1986; Li et al., 2003; Zhang et al., 2012), (2) Zn-deficiency-induced P overaccumulation (Cakmak and Marschner, 1986; Marschner and Cakmak, 1986; Huang et al., 2000; Bouain et al., 2014; Santos et al., 2018) and (3) P-deficiency-induced Zn overaccumulation (Haldar and Mandal, 1981; Misson et al., 2005; Barben et al., 2010; Pedas et al., 2011). The consequences of these P-Zn interactions are negative effects on the yield, quality and longevity of crops and their products with potential negative effects on mineral element composition of produce. This indicates that agricultural management strategies should seek for balanced mineral nutrition to optimize yields and resource use efficiencies.

A prerequisite for the development of crops with greater PUE without reduced Zn concentrations in edible parts is an understanding of the regulatory mechanisms underlying P and Zn deficiency responses and their within-plant interactions. Since P deficiency is perceived by the root tip (Ham et al., 2018) and the subsequent biochemical and morphological adjustments made by plants to increase both P_i_ and Zn acquisition take place in roots, roots have been the focus of research on the molecular responses to P and Zn deficiency. Since acclimation to low P_i_ or Zn supply relies fundamentally on changes in gene expression, recent technical advances in “bottom-up” omics technologies have allowed novel and integrative insights into these processes (Lan et al., 2015). However, many inconsistent observations still need to be resolved before meaningful conclusions can be drawn. For example, although transcriptomic studies have uncovered a surprisingly large number of P_i_ starvation inducible (PSI) genes, these do not respond similarly in all studies, which might be attributed to stochastic fluctuations in gene expression, differences in growth conditions, plant growth stage or tissue sampled, plant nutritional status, differences in sample handling or experimental design, plant species studied, or combination of these factors (Lan et al., 2015). Nonetheless, by combining four studies (Misson et al., 2005; Morcuende et al., 2007; Lan et al., 2012; Woo et al., 2012) of a genetic model plant *Arabidopsis thaliana* (L.) Heynh., 95 core PSI genes were identified, with genes related to lipid metabolism and galactolipid biosynthesis representing the largest group, emphasizing the importance of these processes for P recycling (Lan et al., 2015). Indeed, use of the promoter for SQD1, a gene involved in sulfolipid synthesis, was suggested for the development of “smart plants” to monitor plant P status (Hammond et al., 2003). Further important PSI genes were SPX domain-containing transcription factors, P_i_ transporters of the PHT family and the PHOSPHATE TRANSPORTER TRAFFIC FACILITATOR (Lan et al., 2015). Similarly, these groups of genes have been observed in responses of other plants to P_i_ deficiency for example in *Brassica rapa* L. (Hammond et al., 2011), rice (*Orzya sativa* L.; Secco et al., 2013), maize (*Zea mays* L.; Sun et al., 2016) and poplar (*Populus x canescens* (Aiton) Sm.; Kavka and Polle, 2017) indicating some consensus can be achieved.

In a species-wide study of *Brassica oleracea* L., which has been bred into a wide range of crops such as broccoli, cabbage, kale and cauliflower that are among the most commonly consumed and economically important vegetables in the world (Thomson et al., 2007), large variability in PUE was found that correlated with root development and architecture (Hammond et al., 2009). Concurrently, large genetic variation in shoot Zn concentrations was observed and shoot Zn concentration was found to be increased by increasing soil Zn concentration and reduced by increasing soil P concentrations (Broadley et al., 2010). In contrast, increasing soil P concentration increased shoot calcium (Ca) and magnesium (Mg) concentrations (Broadley et al., 2008). There was no correlation between shoot Zn concentration and shoot DW (i.e. no dilution effect), which indicated that shoot Zn concentration in leafy *Brassica* crops could be improved through cultivar selection. This was recently confirmed for cabbage and broccoli (White et al., 2018). However, a negative correlation between shoot P concentration and shoot DW was observed (Broadley et al., 2010), which indicated that optimal yield required fine tuning P-fertilizer application. Since *Brassica* vegetables are widely consumed and have relatively large Zn concentrations but small concentrations of phytate, which inhibits Zn absorption in the human intestine, they are promising candidates for delivering more bioavailable Zn to human and animal diets (White and Broadley, 2011; White et al., 2018).

In order to understand the early responses of plants to low P and/or Zn supply, differences in gene expression were studied in young roots of two *B. oleracea* accessions grown in a hydroponic system. Plants were analyzed at a time point at which neither ionomic changes nor growth parameters were greatly affected by the treatments. The aim of the study was to identify root-specific molecular networks and genes involved in the responses of the two *B. oleracea* accessions to contrasting P and Zn supply.

## Material and Methods

### Plant Material, Experimental Setup and Ionome Measurements

Seeds of eight *Brassica oleracea* accessions (**Table S1**) with contrasting PUE and shoot Zn concentration (Hammond et al., 2009; Broadley et al., 2010) were obtained from the Genetic Resource Unit, Warwick Crop Centre of the University of Warwick. The accessions showed 3.9-fold variability in PUE (inferred as physiological P efficiency ratio (PPUE) calculated by dividing shoot yield by the shoot P concentration) and 8-fold variability in shoot Zn concentration when plants were grown on a peat-based compost with low P (5.25 mg L^-1^) amendments (Hammond et al., 2009; Broadley et al., 2010). When the same accessions were grown on a peat-based compost with high P (15.75 mg L^-1^) amendments the PPUE varied 14-fold and shoot Zn varied 2.9-fold (Hammond et al., 2009; Broadley et al., 2010). Accessions were grown to maturity in a glasshouse to multiply seeds for subsequent experiments to ensure comparable seed characteristics.

Two independent hydroponic experiments were performed. The phytoavailability of Zn and P in a hydroponic system is generally controlled by fewer factors than in solid substrates, making it the method of choice to study Zn-P interactions (Santos et al., 2018), especially when roots must be harvested (Nguyen et al., 2016). For both experiments, seeds were germinated on the surface of filter paper moistened with deionized water in Petri dishes placed at 16°C in the dark for five days. Six 5-day-old seedlings per accession were transferred to a 5-L bucket holding a solution containing 2 mM Ca(NO_3_)_2_, 2 mM NH_4_NO_3_, 0.75 mM MgSO_4_, 0.5 mM KOH, 0.1 mM FeNaEDTA, 30 µM H_3_BO_3_, 25 µM CaCl_2_, 10 µM MnSO_4_, 3µM CuSO_4_, and 0.5 µM Na_2_MoO_4_, and either 0.25 mM KH_2_PO_4_ (*High P* treatment) or 0.025 mM KH_2_PO_4_ plus 0.225 mM KCl (*Low P* treatment). Either 1 µM ZnSO_4_ (*High Zn*) or no ZnSO_4_ (*Low Zn*) was added to each P treatment. Plants were, therefore, subjected to four treatments (*Low P & Low Zn*, *Low P & High Zn*, *High P & Low Zn*, and *High P & High Zn*). The pH was adjusted to 6 in all treatments by addition of H_2_SO_4_. Experiments were performed in a glasshouse at the James Hutton Institute (Invergowrie, Scotland, UK, latitude 56°27’22.7” N, longitude 03°04’09.9” W) and lasted two weeks, with one change of the nutrient solution, which was continuously aerated. Buckets were set in a complete randomized design on a single bench. Daytime and night time temperatures were maintained at 24°C and 18°C, respectively. Daily irradiance (>200 W m^-2^ for 16 h) was obtained by artificial illumination (MASTER SON-T PIA Green Power lamps, Philips, Guildford, UK).

When the first experiment, which comprised eight *B. oleracea* accessions grown under the said four treatments, was harvested, roots and shoots were separated and dried in an oven at 70°C for three days, before their dry weight (DW) was determined. Six individuals of each accession were subjected to each treatment. Because the root DW was small, roots of 2-3 individuals had to be pooled to obtain one sample for ionome assessment.

For the second experiment, two accessions (C6 and F103) were selected because they have different root architecture and they differ in the amount and the number of root exudates (Pongrac et al. unpublished results). Twenty-four individuals per accession were grown with each of the aforementioned treatments. At harvest, roots of three individuals per accession and per treatment were frozen in liquid nitrogen for RNA extraction yielding three replicates for gene expression analysis. Shoots of these three plants were dried in an oven at 70°C for three days to determine shoot DW. The remaining individuals were photographed (**Figure S1**), root length was determined using a ruler, roots were separated from shoots, dried in an oven at 70°C for three days and weighed. Because the root DW was small, roots of 2-3 individuals were pooled for ionome assessment, yielding 5 to 9 replicates for ionome analysis.

Concentrations of sodium (Na), Mg, P, sulphur (S), chlorine (Cl), potassium (K), Ca, manganese (Mn), nickel (Ni), copper (Cu), iron (Fe), and Zn were determined in plant material using inductively-coupled-plasma-mass spectrometry following microwave-assisted acid digestion of dried roots and shoots as described by White et al. (2012).

### RNA Extraction and RNAseq

Frozen root samples were ground to powder using a pre-cooled pestle and mortar, after which the powdered material was transferred to a 2 ml Eppendorf tube. One mL of Trizol^®^ was added to each sample, shaken thoroughly and incubated at room temperature for 5 min. Subsequently 200µl chloroform was added to each sample after which they were again shaken and incubated for 5 min at room temperature. The samples were centrifuged at 12000×*g* for 15 min at 4°C and 600µl supernatant was transferred to a new 2 ml Eppendorf tube to which 600µl of isopropanol was added. The Eppendorf tubes were inverted for mixing after which samples were centrifuged at 12000×*g* for 15 min at 4°C. The supernatant was removed and the pellet was rinsed in 700µl 70% ethanol and dried at room temperature for 10 min before being resuspended in 100 µl of MiliQ water. The RNA was further purified using the ENZO kit (Zymo Research, Irvine, California, USA) following the manufacturer’s instructions. An “On column DNAse treatment” was included and DNA was eluted twice with 100µl of pre-warmed MilliQ water. Library preparation, single-end reads of 50 bases (SE50) sequencing, data filtering and cleaning, alignments with the reference database (ftp://ftp.ensemblgenomes.org/pub/plants/release-35/fasta/brassica_oleracea/dna/) and gene expression quantification as well as detection of differential gene expression was performed by BGI (https://www.bgi.com/global/) according to their standard analysis pipeline (**Figure S2**). In brief, mRNA, selected *via* polyA tails, was used for cDNA library construction on which SE50 sequencing was performed. Raw reads were filtered to remove adaptor sequences, low quality reads and reads with high content of unknown bases. Sequences were then aligned using Bowtie2 and HISAT tools. Gene expression was quantified as FPKM, i.e. values which consider the gene length and total number of mapped reads and therefore eliminate the influence of gene size differences and sequencing discrepancy on gene expression, using RSEM package. Differentially expressed genes (DEGs) were defined using the NOISeq method (Tarazona et al., 2011) and were identified by comparing the gene expression in the each of the three treatments with the gene expression in the *Low P* & *Low Zn* treatment. NIOSeq uses gene expression values (FPKM) to calculate log2 (fold change) and absolute differences between all comparisons to build a noise distribution model. The average expression in all three replicates was then used to assess if the average fold change and average absolute difference value are above the noise and, if so, the gene was defined as a DEG. A filter of fold change >2 and a diverge probability greater than or equal to 80% were applied. Differentially expressed genes were subsequently mapped to gene ontology (GO) terms (http://www.geneontology.org). In the standard analysis pipeline of BGI a hypergeometric test was used to find significantly enriched GO terms based on “GO Term Finder” (http://www.yeastgenome.org/help/analyze/go-term-finder). The calculated p-values took into account the number of genes belonging to a GO term, the number of DEGs in total, the number of genes for the GO term in the Brassica genome and the overall number of GO annotatable genes. The resulting value was thus scaled for the GO-group size. Additionally, the p-values were Bonferroni corrected for multiple comparisons. Gene ontology terms with p ≤ 0.05 were selected for interpretation.

### Statistical Analysis

The experiments followed a completely randomized design with four treatments and eight accessions in the first experiment and four treatments and two accessions in the second experiment. Figures were created and statistical analyses were performed in RStudio Version 1.0.143 (www.rstudio.com) in combination with R version 3.4.0 (2017-04-21); for graphics the ggplot2 package was used. Using the lsmeans package a two-way analysis of variance (ANOVA) was performed with accession and treatment as independent variables followed by a Tukey post-hoc test at p < 0.05 to assess the effect of accessions and P and Zn supply on biomass and ionome of roots and shoot. For the data in the first experiment, one-way ANOVA followed by a Tukey post-hoc test at p < 0.05 was used to detect significant treatment effects for each of the eight accessions. For two-group comparisons, Student t-test at p < 0.05 was performed. Correlations between shoot dry weight, P and Zn concentration were evaluated using the geom_smooth function of the ggplot2 package method and applying the lm (linear mixed model) method. The ggpmisc package was used to calculate and add p-values and R^2^ values to the graph using the functions stat_poly_eq and stat_fit_glance. Principal component analysis (PCA) was performed using the R packages FactoMineR and factoextra. Data were centred and scaled, and normal distribution confirmed, before principal components (PCs) were computed. Contribution of variables to the computed PCs was depicted graphically using the fviz_contrib function. The individual data points were plotted using the fviz_pca_ind function. Hierarchical clustering was performed using the hcut function of the factoextra package.

## Results

### Growth and Ionome of *Brassica oleracea* Accessions Receiving Contrasting Combinations of P and Zn Supply in Hydroponics

**Figure 1:**
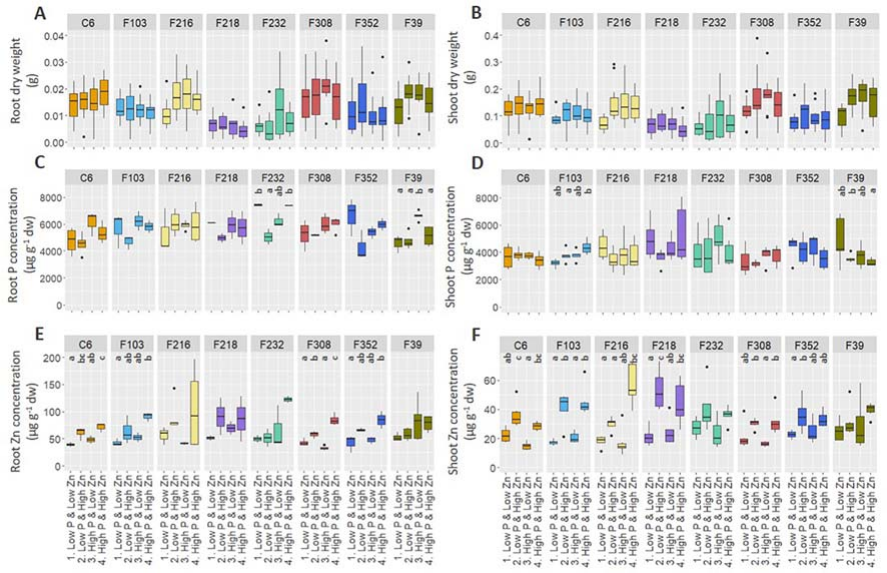
Effect of contrasting combinations of P and Zn supply on eight *B. oleracea* accessions. Plants were grown hydroponically for two weeks after which biomass **(A**, **B)** and P **(C**, **D)** and Zn **(E**, **F)** concentration were determined in roots **(A**, **C**, **E)** and shoots **(B**, **D**, **F)**. Shown are Tukey boxplots representing 25th and 75th percentile of the data with the centerline representing the median, whiskers representing the 5th and 95th percentile and the black dots representing outliers (n = 4-6 for each accession and each treatment). Significant differences between treatments for particular accession are indicated by different letters above the box-plots (one-way analysis of variance followed by Tukey post-hoc test at p < 0.05). dw, dry weight.

Biomass production and element concentrations of roots and shoots were evaluated in eight *B. oleracea* accessions grown hydroponically in solutions with contrasting combinations of P and Zn supply (**Figure 1**). Considering genotype and treatment as independent variables, two-way ANOVA (**Table 1**) revealed that, for the eight accessions studied: (1) genotype affected root length and shoot P concentration, but treatment and genotype × treatment interactions did not, (2) both genotype and treatment affected root and shoot DW, but their interaction did not, (3) genotype, treatment and their interaction affected root P concentration and shoot Zn concentration, and (4) treatment affected root Zn concentration, but genotype and genotype × treatment interaction did not. Since genotype × treatment interactions were significant only for some responses, one-way ANOVA was conducted to depict treatment effects for each accession separately. No treatment effects were observed on root or shoot biomass (**Figures 1A, B**). No apparent effects of P supply on root and shoot Zn concentrations were found in the *B. oleracea* accessions studied (**Figures 1E, F**). Zinc concentration increased with increasing Zn supply in both roots and shoots of all accessions grown hydroponically in the experiments reported here (**Figures 1E, F**).

**Table 1:** Two-way analysis of variance table with p-values (those less than 5% are highlighted in bold) and means squares (in italics) for response variables in eight *B. oleracea* accessions grown in hydroponics at *Low P* and *High P* each with *High Zn* or *Low Zn* treatments for two weeks (n = 4-6 for each accession and each treatment).

Parameter	Genotype	Treatment	Accession x Treatment
**Root length**	**<0.001**	0.494	0.825
	*2.34E+04*	*3.24E+03*	*2.87E+03*
**Root dry weight**	**<0.001**	**0.019**	0.302
	*7.62E-04*	*1.51E-04*	*5.17E-05*
**Shoot dry weight**	**<0.001**	**<0.001**	0.308
	*4.78E-02*	*3.06E-02*	*3.34E-03*
**Root P concentration**	**0.012**	**<0.001**	**0.038**
	*2.13E+06*	*6.48E+06*	*1.34E+06*
**Shoot P concentration**	**0.018**	0.18	0.135
	*2.43E+06*	*1.59E+06*	*1.33E+06*
**Root Zn concentration**	0.212	**<0.001**	0.48
	*8.49E+02*	*7.95E+03*	*5.97E+02*
**Shoot Zn concentration**	**0.001**	**<0.001**	**<0.001**
	*2.86E+02*	*4.12E+03*	*2.28E+02*

**Figure 2:**
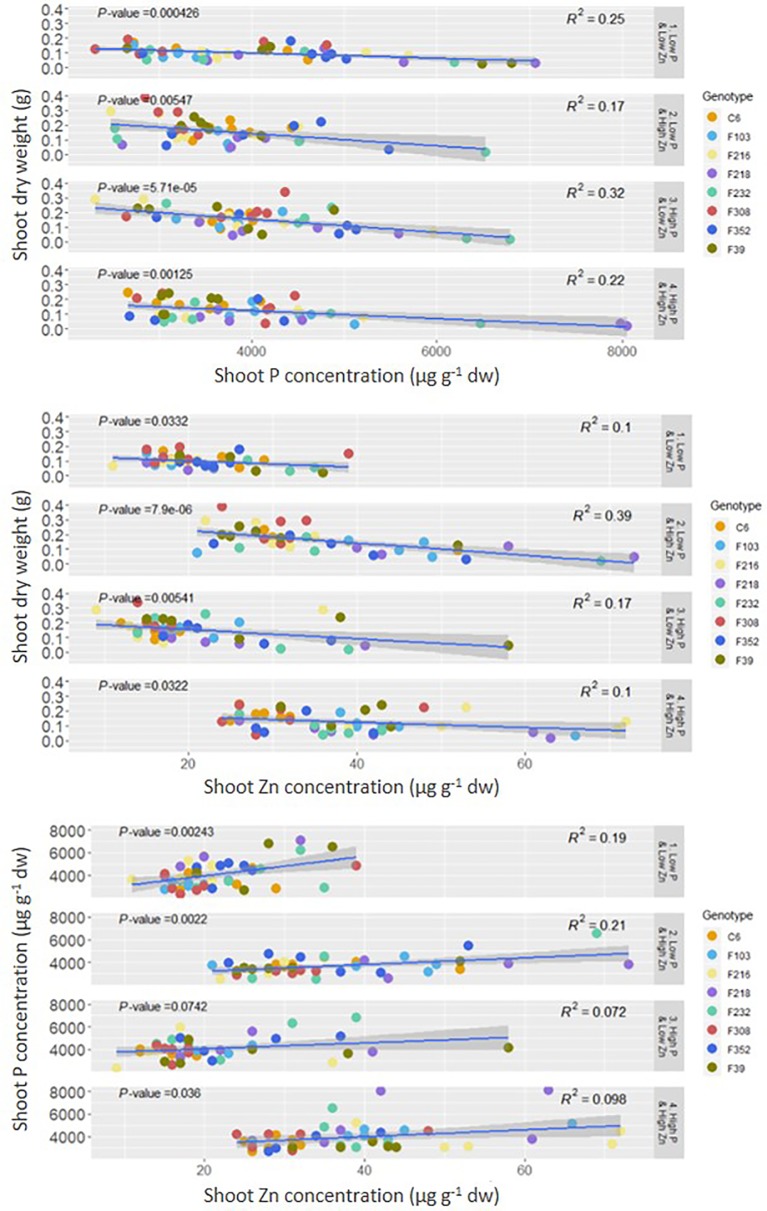
Correlation between shoot P or Zn concentration and shoot dry weight and between shoot P and Zn concentration in eight *B. oleracea* accessions grown hydroponically for two weeks with contrasting combinations of P and Zn supply. Significant correlations are indicated by p-value < 0.05 dw, dry weight.

Both shoot P concentration and shoot Zn concentration were negatively correlated with shoot DW, while there was a positive correlation between shoot P concentration and shoot Zn concentration (**Figure 2**).

### Responses of the C6 and the F103 Accessions Receiving Combinations of P and Zn Supply in Hydroponics

The F103 accession was selected for further detailed analysis because of its marked response to the nutritional treatments in the first experiment (i.e. treatment effects were observed for shoot P and Zn concentrations and root Zn concentrations, **Figures 1D–F**). The C6 accession was selected because its shoot Zn concentration, but not its shoot P concentration, was affected by the nutritional treatments in the first experiment (**Figures 1C–F**). In addition, these two accessions differed in their root architecture with the F103 accession having a larger number of lateral roots, which were also longer than in the C6 accession, and in their root exudation with the C6 accession releasing a larger quantity and number of polar compounds than the F103 accession (Pongrac et al., unpublished results).

**Figure 3:**
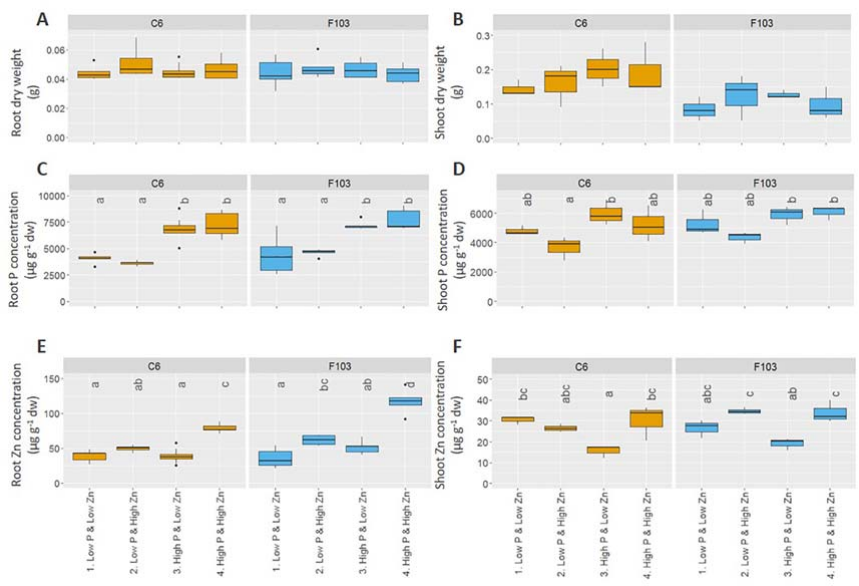
Effect of contrasting combinations of P and Zn supply on two *B. oleracea* accessions (C6 and F103). Plants were grown hydroponically for two weeks after which biomass **(A, B)** and P **(C, D)** and Zn **(E, F)** concentration were determined in roots **(A, C, E)** and shoots **(B, D, F)**. Shown are Tukey boxplots representing 25th and 75th percentile of the data with the centerline representing the median and whiskers representing the 5th and 95th percentile (n = 5-9 for roots and n = 3 for shoots for each accession and each treatment). Significant differences between accessions and treatments are indicated by different letters above the box-plots (Two-way analysis of variance followed by Tukey post-hoc test at p < 0.05). dw, dry weight.

Root length, DW and ionome (concentrations of P, Zn, Mg, S, Cl, K, Ca, Mn, Fe, Ni, Cu, and Na) of roots and shoots, and root gene expression were evaluated in the two selected accessions. Across all treatments, roots of *B. oleracea* accumulated less biomass (**Figure 3**) and had smaller concentrations of Mg, S, Cl, K, Ca, Mn, and Ni than shoots (**Figures S3** and **S4**). The two-way ANOVA, considering the genotype and treatment as independent variables (**Table 2**), revealed that the genotype affected root and shoot DW, while the treatment and genotype × treatment interactions did not. In agreement with the first experiment, the C6 accession had, on average across all treatments, significantly larger roots and shoots than the F103 accession, but no treatment-specific differences were observed for root and shoot DW of the two accessions (**Figures 3A, B**). Only the treatment, but not the genotype or genotype × treatment interactions, affected root and shoot P concentrations and shoot Zn concentration (**Table 2**). An increase in P supply increased root P concentration in both accessions, while in shoots no significant effects of P and Zn supply were seen (**Figures 3C, D**). An increase in Zn supply increased root Zn concentrations, except in the C6 accession at *Low P* supply (**Figure 3E**). The largest root Zn concentration was found in the *High P & High Zn* treatment (**Figure 3E**) in both accessions. In shoots, Zn concentrations increased significantly with increasing Zn supply in both accessions at *High P*, but not at *Low P* (**Figure 3F**).

**Table 2:** Two-way analysis of variance table with p-values (those less than 5% are highlighted in bold) and means squares (in italics) for response variables in two *B oleracea* accessions (the C6 and the F103) grown in hydroponics at *Low P* and *High P* each with *High Zn* or *Low Zn* treatments for two weeks (n = 3 for each accession and each treatment).

Parameter	Genotype	Treatment	Accession x Treatment
**Root length**	**0.032**	**<0.001**	**0.025**
	*1.29E+01*	*5.36E+01*	*9.60E+00*
**Root dry weight**	**0.024**	0.495	0.275
	*4.17E-04*	*5.56E-05*	*9.44E-05*
**Shoot dry weight**	**0.005**	0.445	0.819
	*2.91E-02*	*2.59E-03*	*8.48E-04*
**Root P concentration**	0.094	**<0.001**	0.568
	*1.13E+06*	*1.89E+07*	*2.47E+05*
**Shoot P concentration**	0.14	**0.002**	0.738
	*3.18E+06*	*3.81E+07*	*2.98E+05*
**Root Zn concentration**	**<0.001**	**<0.001**	**<0.001**
	*1.28E+03*	*4.46E+03*	*5.22E+02*
**Shoot Zn concentration**	0.112	**<0.001**	0.145
	*5.13E+01*	*2.69E+02*	*3.75E+01*

**Figure 4:**
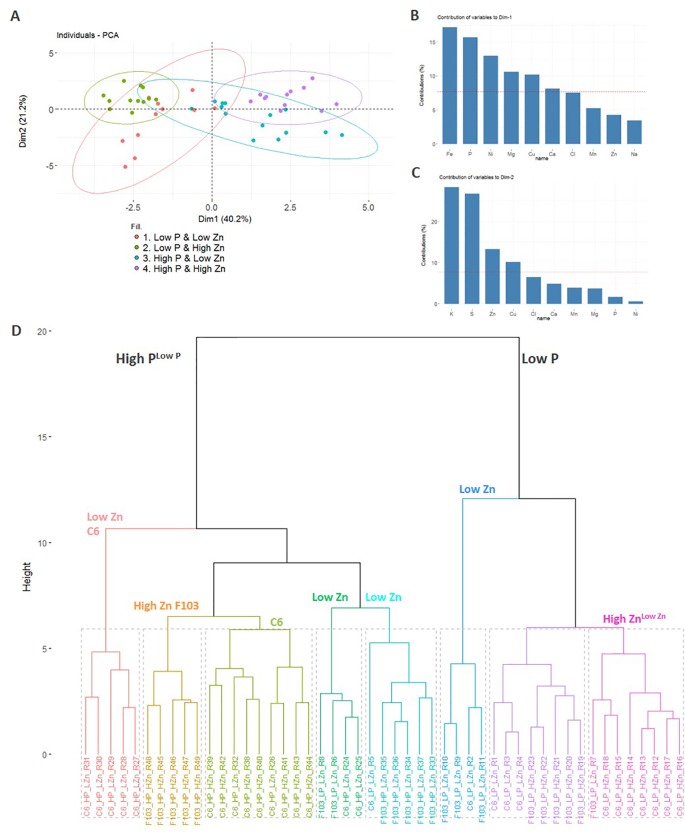
Principal component analysis (PCA) of the root ionome of two *B. oleracea* accessions (C6 and F103). The root dry weight and concentrations of Na, Mg, P, S, Cl, K, Ca, Mn, Fe, Cu, Zn and Ni in roots of the C6 and F103 accessions grown hydroponically for two weeks at different P and Zn supply were included in the PCA to detect major effects on variations observed. The PCA plot depicts the first two PCs (Dim1 and Dim2) for response variables grouped by treatments (**A**). The bar plots show the contribution of ten variables to PC1 **(B)** and PC2 **(C)**. Cluster dendrogram of all replicates (n = 4-9 for each accession and each treatment; (**D**). In (**B** and **C**), the red dashed line at 7.7% indicates the value at which uniform contribution of all variables tested occurs.

A PCA was used to visualize the major sources of variation in root and shoot ionomes of the two accessions. Root and shoot samples separated clearly and a greater spread of data were observed in the root ionome than in the shoot ionome (**Figure S5**). A detailed PCA of the root ionome based on DW, P and Zn concentrations (**Figures 3C–F**), Mg, S, Cl, K and Ca concentrations (**Figure S3**) and Mn, Fe, Ni, Cu and Na concentrations (**Figure S4**) revealed that the first two PCs accounted for 61.4% of the variance (**Figure 4A**). In the PCA plot of the roots of individual plants a clear separation of the ionome was seen between *Low P* and *High P* treatments in the treatments with *High Zn*. By contrast, in the treatments with *Low Zn* the P supply did not lead to a clear effect on the root ionome. Variables with significant contributions to the first PC were root Fe, P, Ni, Mg, Cu and Ca concentrations (**Figure 4B**), while variables with significant contribution to the second PC were root K, S, Zn and Cu concentrations (**Figure 4C**). Root P and Fe concentrations were significantly smaller in plants receiving a *Low P* supply than those receiving a *High P* supply (**Figures 3C** and **S4C**).

Independently, in a hierarchical clustering analysis of the root ionome, samples separated clearly depending on the P supply (**Figure 4D**). Within the *High P* cluster, the majority of samples were grouped based on the Zn supply. Within the *High P* cluster, samples of the F103 accession from the *High Zn* treatment formed a group, as did those from the *Low Zn* treatment, indicating a genotypic effect. Notably, the P supply had a stronger influence on the clustering of samples than the Zn supply, which in turn had a greater effect on clustering than did the genotype, whose effects were not consistent. In order to link these observations and expand our understanding of P-Zn interactions in *B. oleracea* gene expression and regulatory networks in roots were studied in greater detail.

### Gene Expression of the *Brassica oleracea* C6 and F103 Accessions Receiving Combinations of P and Zn Supply in Hydroponics

Roots of the C6 and the F103 accessions receiving four combinations of P and Zn supply for two weeks in hydroponics were analyzed *via* RNAseq in triplicates (all genes can be found in **Table S2**). Raw data of the RNAseq experiment can be downloaded from the Gene Expression Omnibus (https://www.ncbi.nlm.nih.gov/geo/) database, accession number GSE127467. Sequencing of 24 samples returned on average 23,569,302 clean reads per sample (72.2%) which were then mapped to the reference genome (ftp://ftp.ensemblgenomes.org/pub/plants/release-35/fasta/brassica_oleracea/dna/), while the average genome mapping ratio was 91.78%, showing that on average 91% of all reads could be mapped to the reference genome. On average the expression of 38,103 genes was detected.

### Treatment Effects on the Patterns in Gene Expression in the Two *B. oleracea* Accessions

**Figure 5:**
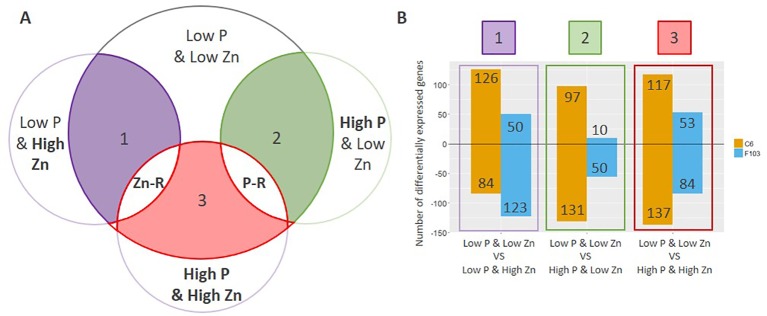
Comparative analysis of differentially expressed genes (DEGs) in roots of *B. oleracea* accessions C6 and F103 grown hydroponically for two weeks with contrasting combinations of P and Zn supply. Differentially expressed genes were defined by comparing the *Low P & Low Zn* treatment with the three other treatments as schematically represented in **(A)**, where the contrasting supply (Low vs. High) is highlighted in bold. The number of DEGs (up-regulated are positive values and down-regulated are negative values) in each of these three comparisons for each accession is displayed in **(B)**. Genes that were either consistently up-regulated or down-regulated in response to either Zn or P supply in both or only in one of the accessions were defined as Zn-responsive (Zn-R) and P-responsive (P-R).

To understand the way by which contrasting combinations of P and Zn supply affect gene expression in *B. oleracea*, differentially expressed genes (DEGs) were identified. The expression of genes in roots of plants receiving *Low P & High Zn*, *High P & Low Zn* or *High P & High Zn* supply was compared to that in roots of plants receiving *Low P & Low Zn* (**Figure 5A**, comparison 1 through 3). All details regarding the comparisons are provided in **Table S3**. In general, a larger number of DEGs were found in the C6 accession than in the F103 accession (**Figure 5B**).

**Figure 6:**
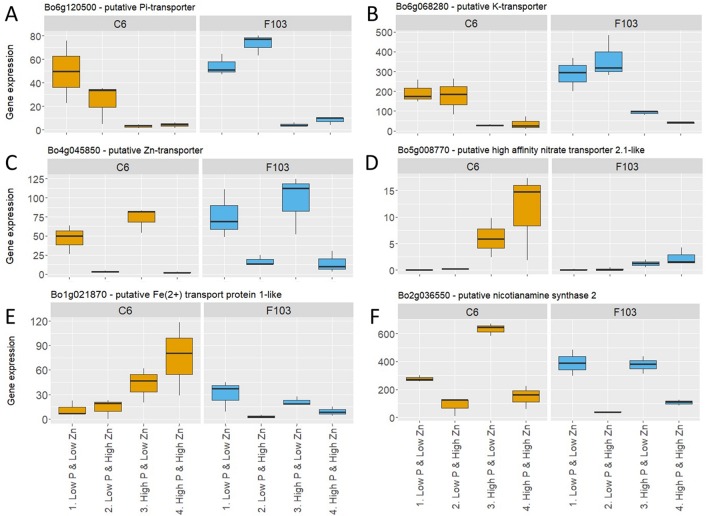
Expression of selected P-responsive and Zn-responsive genes in roots of *B. oleracea* accessions C6 and F103 grown hydroponically for two weeks with contrasting combinations of P and Zn supply. These genes were either P-responsive in both accessions: **(A)** Pi-transporter (Bo6g120500) and **(B)** K-transporter (Bo6g068280), Zn-responsive in both accessions: **(C)** putative Zn-transporter (Bo4g045850), P-responsive in the C6 accession: **(D)** predicted high-affinity nitrate transporter 2.1-like (Bo5g008770) and **(E)** putative iron^2+^ transport protein1-like (Bo5g008770), or Zn-responsive in the F103 accession: **(F)** predicted nicotianamine synthase 2 (Bo2g036550).

Following the selection of DEGs in different treatments, P-responsive and Zn-responsive (P-R and Zn-R, respectively) genes were defined (**Figure 5A**). A gene was considered to be P-responsive if it responded to *High P* treatment (expression in the *Low P & Low Zn* treatment compared to expression in the *High P & Low Zn* treatment and compared to expression in the *High P & High Zn* treatment) with either an increase (up-regulated) or decrease (down-regulated) in expression. Likewise, Zn-responsive genes were defined as those responding to increased Zn supply (expression in the *Low P & Low Zn* treatment compared to expression in the *Low P & High Zn* treatment and compared to expression in *High P & High Zn*). Twenty-seven P-responsive genes were detected in the two accessions. Of these 26 were down-regulated and one was up-regulated. Among the down-regulated P-responsive genes was a putative P_i_-transporter (Bo6g120500, orthologue of AT1G20860: Probable inorganic phosphate transporter 1-8), whose expression was 6 to 16-fold smaller in the *High P* treatments (**Figure 6A**). Another gene down regulated in both treatments with *High P* was a predicted AtHAK5 homologue (**Figure 6B**), a high-affinity K-transporter (White, 2013), whose expression changes were not reflected in changes in root or shoot K concentrations (**Figures S3G**, **H**). Also common to both accessions responding to increased Zn supply was the down-regulation of four Zn-responsive genes, one of which was a predicted Zn transporter from *B. oleracea* var. *oleracea* (Parkin et al., 2014), whose expression was repressed under *High Zn* (**Figure 6C**).

A larger number of P- and Zn-responsive genes were unique to only one accession. Ninety-two P-responsive genes, 55 of which were down-regulated and 37 up-regulated by increasing P supply, were found in the C6 accession, while in the F103 accession only six P-responsive genes were found, of which two were down-regulated and four were up-regulated by increasing P supply. An example of a P-responsive gene in the C6 accession encoded a predicted high-affinity nitrate transporter 2.1-like protein. This gene was induced at *High P & Low Zn* and at *High P & High Zn* when compared to *Low P & Low Zn* (**Figure 6D**). A second example of a P-responsive gene in the C6 accession only was a putative iron^2+^ transport protein1-like, whose expression was induced in treatments with *High P* supply compared to the treatments with *Low P* supply (**Figure 6E**). Thirty-four Zn-responsive genes were found in the C6 accession, of which seven were down-regulated and 27 were up-regulated by increased Zn supply, while in the F103 accession 28 Zn-responsive genes were found of which 21 were down regulated and seven were up-regulated by increased Zn supply. An example of a Zn-responsive gene in the F103 accession encoded a predicted nicotianamine synthase (NAS) 2, whose expression was reduced by increased Zn supply (**Figure 6F**).

### Gene Ontology Analysis

**Figure 7:**
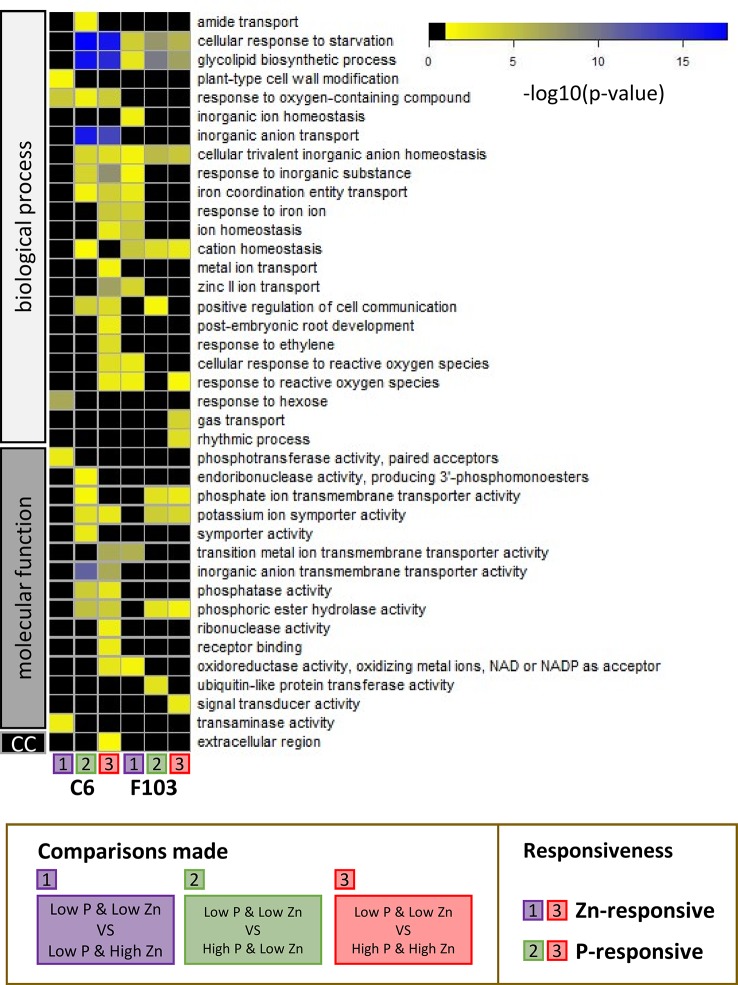
Gene ontology (GO) analysis of enriched functional categories in roots of *B. oleracea* accessions C6 and F103 grown hydroponically for two weeks with contrasting combinations of P and Zn supply. Differentially expressed genes in the C6 and the F103 accessions were defined between the treatments *Low P & Low Zn* with *Low P & High Zn* [1], with *High P & Low Zn* [2] and with *High P & High Zn* [3] for each accession. See **Figure 5A** for schematic representation of comparisons. Among these DEGs the GO-term enrichments were calculated and scored by Bonferroni corrected p-values as indicated by the color legend (in black: GO-term with p-values above 0.05, yellow to blue: increasing significance for GO-enrichment). Gene ontology term enrichments in the GO-groups: biological process, molecular function and cellular compartment (cc) were detected. Where child and ancestral GO-categories where enriched, only the most defined child categories were selected.

To capture the physiological processes involved in responses to contrasting combinations of Zn and P supply, a GO term enrichment analysis was performed. For ease of discussion, GO term enrichments among DEGs were labeled as follows: responsive to Zn supply (comparisons 1 and 3) and responsive to P supply (comparisons 2 and 3) in either the C6 or the F103 accession. All GO terms with significant p-values are shown in **Table S4**. Thirty-nine significantly enriched GO term categories were found, which belonged to groups annotated to biological processes, molecular function or cellular compartment (**Figure 7**). In response to Zn supply there was a GO-term enrichment in “response to oxygen containing compound” in the C6 accession, while there was a GO-term enrichment in “cellular response to starvation,” “glycolipid biosynthetic process,” “cellular trivalent inorganic anion homeostasis,” “cation homeostasis” and “response to reactive oxygen species (ROS)” in the F103 accession. None of the enrichments were common to both accessions in response to Zn supply, while five processes (“cellular response to starvation,” “glycolipid biosynthetic process,” “cellular trivalent inorganic anion homeostasis,” “potassium symporter activity” and “phosphoric ester hydrolase activity”) were enriched in both accessions as a response to changing P supply (**Figure 7**), showing a conserved transcriptional response to vagaries in P supply. In addition, accession-specific GO enrichments were found. Only one process (“response to oxygen-containing compound”) was common to both Zn and P responses (significant enrichment in all three comparisons) in the C6 accession while four such processes (“cellular response to starvation,” “glycolipid biosynthetic process,” “cellular trivalent inorganic anion homeostasis” and “cation homeostasis”) were common to both Zn and P responses in the F103 accession.

## Discussion

### Growth and Ionome Responses of *B. oleracea* Accession Receiving Combinations of P and Zn Supply in Hydroponics

To understand the molecular networks and genes involved in the early responses of *B. oleracea* to contrasting P and Zn supply, gene expression in roots of young plants was profiled in a hydroponic system. Two *B. oleracea* accessions, C6 and F103, which responded significantly to the contrasting P and Zn supply (**Figure 1**) and differ in their root architecture and profiles of root exudates (Pongrac et al., unpublished results) were selected from a set of eight accessions (**Table S1**) for further analysis. The hydroponic experiments were designed to capture early root responses at a molecular level, with minimal effects on plant growth and tissue ionomes. There were no interactions of treatments and genotypes for shoot biomass or shoot P concentration (**Tables 1** and **2**) and the shoot P concentration (**Figures 1D** and **3D**) was above the shoot P concentration reported to be sufficient (2,000 mg P kg^-1^ DW) in *B. oleracea* (Campbell, 2000). Purple anthocyanin pigmentation, which is indicative of systemic P deficiency, was also absent (**Figure S1**; Jiang et al., 2007). Additionally, in plants grown in treatments with *Low Zn* supply, shoot Zn concentrations were at the lower limit of Zn sufficiency, which is reported to be 20 mg Zn kg^-1^ DW for *B. oleracea* (Campbell, 2000), but were never significantly below this value (**Figures 1F** and **3F**). Two explanations for the lack of deficiency symptoms observed in these young Brassica plants can be offered. The first is that seed reserves, which may provide sufficient P supply from one to four weeks (White and Veneklaas, 2012), could have provided adequate nutrition of plants. A delay in reducing growth in young plants challenged with P and Zn deficiencies has been reported previously for barley (Huang et al., 2000). The second explanation is that differences at the molecular level in roots of plants grown in different treatments, enable the activation of regulatory networks compensating for insufficient P or Zn supply. Large differences in gene expression were observed in roots of both *B. oleracea* accessions in response to contrasting P and Zn supply in the hydroponic system (**Figures 5**–**7**). Thus, studying young plants receiving sub-optimal P and Zn supply that do not exhibit visual deficiency symptoms allowed us to capture key compensatory mechanisms enabling optimal growth of these plants at the molecular level.

Plants in the hydroponic system grew well (**Figure S1**) and had similar shoot P concentrations (**Figure 1D**) as *B. oleracea* plants grown on compost (Hammond et al., 2009; Broadley et al., 2010; **Table S1**). By contrast, shoot Zn concentrations (**Figure 1F**) were less when grown in the hydroponic system, even with an adequate Zn supply, than shoot Zn concentrations observed when these accessions grown in compost (Hammond et al., 2009; Broadley et al., 2010; **Table S1**). These results suggest that shoot P concentration is less variable than shoot Zn concentration across growth environments. Similarly, shoot P concentration in cabbage (*B. oleracea* var. *capitata*) showed 3-fold variation, while shoot Zn concentration varied 15-fold, in plants grown on three unamended soil types (Pongrac et al., 2019). Such differences in variation between concentrations of macro- and micronutrients were also reported for *A. thaliana* (Berezin et al., 2012). Noteworthy was a negative correlation between shoot P and Zn concentrations with shoot dry weight, but a positive correlation between shoot P concentration and shoot Zn concentration, among the *B. oleracea* accessions grown in the hydroponic system (**Figure 2**). In line with similar observations in *B. oleracea* grown in compost (Broadley et al., 2010) and in cereals (Murphy et al., 2008; Zhao et al., 2009), these observations reflect the delicate balance between yield and mineral element composition that must be considered carefully in breeding and biofortification programs. Overall, these results indicate the suitability of the hydroponic system for experiments to study gene expression in roots and identify early molecular changes in response to contrasting nutrient supply.

The root system is the plant organ that first responds to changes in the rhizosphere. Therefore, it was not surprising to observe larger variation in the root than in the shoot ionome (**Figure S5**) and a larger number of significant differences between treatments in the concentrations of elements in roots than in shoots (**Figures 3**, **S3**, and **S4**). Most prominent was the greater root Fe concentrations in treatments receiving *High P* supply than those receiving *Low P* supply (**Figure S4C**), while shoot Fe concentrations were hardly affected (**Figure S4D**). This highlights an interesting connection between P and Fe homeostasis that has also been observed in other plant species. In roots of strawberry (*Fragaria × ananassa* (Duchesne ex Weston) Duchesne ex Rozier), for example, Fe concentration was strongly reduced upon P starvation (Valentinuzzi et al., 2015) as was the Fe concentration in rice (*Oryza sativa*) roots (Saenchai et al., 2016) and maize (*Zea mais*) roots (Nichols et al., 2012). By contrast, roots of P-starved *A. thaliana* plants had significantly larger Fe concentrations than did roots of P-replete plants in two different studies (Misson et al., 2005; Baxter et al., 2008; Raghothama et al., 2008), as did roots of bush bean (*Phaseolus vulgaris* L.; Wallace et al., 1974) and rice seedlings (Zheng et al., 2009). No effect of P supply was observed on Fe concentrations in roots of maize (Prabhakaran Nair and Rabu, 1975) or poplar (Kavka and Polle, 2017). These contradicting observations preclude consensus on the interactions between P deficiency and Fe nutrition, but they do indicate crosstalk between Fe and P nutrition which merits further investigation (Briat et al., 2015; Bouain et al., 2019), especially since Fe deficiency in humans is of great concern and Fe fortified food would benefit many of the world’s population (White and Broadley, 2009).

In order to evaluate the source of the variation in the root ionome two independent techniques were used: PCA and cluster analysis. They both showed (**Figure 4**) that P supply had a larger effect on the root ionome than did Zn supply, possibly reflecting a greater importance of P for metabolic processes and plant growth than Zn. Even though the *B. oleracea* accessions studied in the hydroponic system did not show different growth rates in response to contrasting P supply, these would be expected subsequently (Dechassa et al., 2003). Additionally, the PCA analysis revealed root Fe concentration as the strongest contributor (explaining 40.2% of the variation in the root ionome) to PC1, which further stresses the connection between Fe and P homeostasis in plants.

### Gene Expression in Roots of *B. oleracea* Accessions Receiving Different P and Zn Supplies

Significant differences in gene expression in roots of the two *B. oleracea* accessions were observed depending upon P and Zn supply, as reflected by the large number of DEGs (**Figure 5B**). A greater number of DEGs responding to P and Zn supply were observed in the C6 accession than in the F103 accession, which might be linked with the observation that C6 releases a larger number and greater amounts of root exudates than the F103 accession (Pongrac et al., unpublished results). To uncover DEGs responding most significantly P and Zn supply, P-responsive and Zn-responsive genes were defined (**Figure 5A**). Of the 27 P-responsive genes detected in the two accessions, 26 were down-regulated and one was up-regulated. These genes represent the constitutive response of the two *B. oleracea* accessions to P supply. Orthologues of fourteen *B. oleracea* P-responsive genes found in our study could be found within a set of 95 core PSI genes in *A. thaliana* (Lan et al., 2015), illustrating a common genetic response to *Low P* across different species. Among these fourteen genes were two genes encoding P_i_ transporters Bo6g120500 and Bo6g120510, orthologues of At1g76430 or PHT1;9 (P_i_ transporter 1;9), and Bo3g134470, an orthologue of At3g47420 or G3Pp1 (P_i_ starvation-induced gene 3). PHT1;9, together with PHT1;8, plays an important role in P_i_ acquisition during P-starvation (Remy et al., 2012) and G3Pp1 is a member of a gene family of Glycerol-3-phosphate permeases, likely to be involved in the mobilization of G3P (Glycerol -3- phosphate) during P-starvation (Misson et al., 2005). Members of the latter gene family are induced greatly upon P starvation and *A. thaliana* mutants in G3Pp1 show longer lateral roots than wild type indicative of a P-starvation phenotype even under sufficient P supply (Ramaiah et al., 2011).

Among the down-regulated P-responsive genes was a putative P_i_-transporter (Bo6g120500, orthologue of AT1G20860: Probable inorganic phosphate transporter 1-8), whose repressed expression in the *High P* treatments (**Figure 6A**) suggested that this was a high affinity P transporter required for P_i_ uptake at low external P_i_ concentrations. These have been shown to be repressed by an increase in P supply in *A. thaliana* (Mudge et al., 2002; Bayle et al., 2011; Remy et al., 2012) and also to be induced by Zn deficiency in barley (*Hordeum vulgare* L.; Huang et al., 2000).

Both accessions responded to increased Zn supply by down-regulation of four Zn-responsive genes, one of which (Bo4g045850) was a predicted Zn transporter from *B. oleracea* var. *oleracea*, whose expression was repressed under *High Zn* (**Figure 6C**). Bo4g045850 is an orthologue AtZIP3 (Zn transporter precursor), a Zn transporter which is induced by Zn deficiency in *A. thaliana* (Inaba et al., 2015) but requires further investigation to uncover its mode of action. Other members of the ZIP family have been studied in *A. thaliana* and their regulation under Zn deficiency has been demonstrated. ZIP9 and ZIP12 for example play a role in Zn uptake with mutants lacking these transporters showing reduced Zn concentrations (Inaba et al., 2015). This Zn transporter (Bo4g045850) might provide a molecular target for breeding cultivars with larger tissue Zn concentrations, however detailed understanding of its regulation will be needed to ensure its optimal expression for significant increase in Zn uptake and larger shoot Zn concentrations.

Another P-responsive gene in roots of the C6 accession was Bo5g008770, a putative high affinity nitrate transporter and orthologue to AtNRT2.1 (nitrate transporter 2.1). In *A. thaliana*, AtNRT2.1 is induced by N starvation and repressed by treatment with ethylene (Zheng et al., 2013). It has also been implicated in lateral root initiation, which results in more root branching when N supply limits plant growth (Zheng et al., 2013). It was reported that P deficiency can induce ethylene production (Borch et al., 1999), which may explain the observed induction of Bo5g008770 by increased P supply in the C6 accession (**Figure 6D**). It is likely that increasing P supply reduces ethylene production which then allows the induction of Bo5g008770 expression. Thus, the uptake of P and N might be balanced to provide an optimal N:P ratio in the plant (Raven, 2015). Another example of a P-responsive gene in roots of the C6 accession is Bo1g021870, which encodes a solute carrier family 39 (zinc transporter) protein (**Figure 6E**). The *A. thaliana* orthologue is AtIRT1 (Iron-Regulated Transporter 1) which is induced by Fe deficiency and in the presence of Zn, is able to affect the accumulation of different divalent cations such as Fe^2+^, Zn^2+^, Mn^2+^, Cd^2+^ and Cu^2+^ and acts together with IRT2 to maintain Fe homeostasis (Vert et al., 2009; Shanmugam et al, 2011). Since Bo1g021870 was up regulated in roots of accession C6, but not in roots of accession F103, in the *High P* treatment, this might explain in part the large root Fe concentration of the C6 accession under these conditions (**Figure S4C**).

A Zn-responsive gene unique to the F103 accession was Bo2g036550: a predicted nicotianamine synthase and orthologue to AtNAS2, whose expression was reduced by increased Zn supply (**Figure 6F**). It could be linked to the role of nicotianamine in transporting Zn within the plant (White and Broadley, 2011; Deinlein et al., 2012) or in solubilizing Zn in the soil through the synthesis and release of phytosiderophores (Clemens et al., 2013). Just like its *A. thaliana* counterpart, BoNAS2 could have played a role in increasing shoot Zn concentration in plants from the *Low Zn* treatments when its expression was high. An interaction between plant P, Zn and Fe nutrition has been proposed to act *via* PHR1 (Phosphate Starvation Response 1) and PHL1 (PHR LIKE 1) associations with the promoter of Fer1 (Ferritin 1), which allow the expression of Fer1 to be regulated in an Fe-independent manner (Briat et al., 2015). The smaller root Fe concentration in plants from the *Low P* treatment compared to those from the *High P* treatment (**Figure S4C**) might, therefore, be linked to changes in the expression of genes connected with Fe homeostasis, such as BoNAS2 (Bo2g036550). Overexpression of NAS genes has been associated with Fe-deficiency tolerance in several plant species (Nozoye, 2018). NAS1, was one of only two genes consistently downregulated in response to P_i_ deficiency in *A. thaliana* according to four studies analyzing expression in roots or whole seedlings (Lan et al., 2015).

### Gene Ontology Analysis

The greater number of the P-responsive genes in roots of the C6 accession than in roots of the F103 accession suggest that these two accessions might employ different genetic strategies when challenged by an insufficient P supply. The GO enrichment analysis highlighted these differences (**Figure 7**). For example, it revealed an enrichment in “inorganic anion transmembrane transporter activity” in the C6 accession but not in the F103 accession. This could indicate a greater flexibility in ion transport capabilities of the C6 accession. Interestingly, it was observed that GO-terms are less conserved among Zn-responsive genes and depend much more on genotype and P supply (**Figure 7**). This might suggest that the response to Zn supply is less conserved and more varied than the response to P supply. Notably, “Zinc II ion transport” processes were enriched among genes whose expression was changed by Zn supply in the C6 accession only at *High P,* while in the F103 accession this occurred at *Low P* supply.

Other important pathways were the “cellular response to starvation” and “glycolipid biosynthetic process” which were enriched in both accessions under almost all conditions assayed, highlighting the robust changes in response to contrasting combinations of P or Zn supply (**Figure 7**). As has been reported previously, a lack of P leads to remodelling of lipids, which is enabled by changes in gene expression (Nakamura, 2013; Tawaraya et al., 2014; Siebers et al., 2015), particularly through PHR1 in *A. thaliana* (Pant et al., 2015). Phosphate deficiency results in alterations in the membrane lipid composition of plants, because phospholipids are replaced with non-phosphorus glycolipids and sulfolipids (Li et al., 2006). Lipids play crucial roles in the ability of plants to survive and acclimatize to environmental changes. The lipid response during P_i_ deprivation differs between leaves and roots, as leaves contain large amounts of galactolipids (Siebers et al., 2015). Membrane phospholipids constitute about 20% of the total amount of P in the leaves of P_i_-replete plants, and thus represent a large pool of P that can be mobilized when internal P_i_ levels are insufficient to match the demand of the plant (Veneklaas et al., 2012). In many plants, as exemplified by poplar, changes in P availability affect the expression of genes belonging to the functional category “galactolipid synthesis” (Kavka and Polle, 2017).

Taking a closer look at GO category “cellular response to reactive oxygen species,” which was enriched in the P-responsive genes of the F103 accession and also among genes responsive to a combination of P and Zn supply in the C6 accession, suggests that the sensing of P-withdrawal might occur through the formation of ROS, in agreement with previous reports (Ham et al., 2018). Interplay between signaling networks for P deficiency and Zn deficiency through ROS might enable the C6 accession to integrate both signals and ensure an appropriate response to precise environmental conditions.

Taking into consideration that all analysis performed was limited by the quality of the gene annotation of the *B. oleracea* genome, the value of the dataset is likely to increase in the future when more information become available.

## Summary

Early responses to insufficient P and Zn supply were assessed by analyzing gene expression in roots of young *B. oleracea* plants, not yet exhibiting visible deficiency symptoms. A number of Zn and P-responsive genes were detected. Their expression changes were related to acclimation to low-nutrient environments and contributed to maintaining balanced nutrition. The gene expression changes agreed with previous studies demonstrating alterations in P_i_ transport and phospholipid metabolism in response to reduced P_i_ supply. It is anticipated that improved knowledge of genes responsive to P or Zn supply will help illuminate their roles in uptake and accumulation of P and Zn and might identify candidate genes for breeding high-yield-high-Zn brassicas.

## Data Availability Statement

The datasets generated for this study can be found in Gene Expression Omnibus, GSE127467.

## Author Contributions

PP and PW conceived and designed the research. PP, JT, GW, and SF conducted experiments. PP and SF analyzed data. PP, SF, and PW wrote the manuscript. All authors read and approved the manuscript.

## Funding

This work was supported by the Rural and Environment Science and Analytical Services Division of the Scottish Government, an EU Marie Curie Intra-European Fellowship (REA grant agreement n°623305) to PP who also acknowledges financial support from the Slovenian Research Agency (P1-0212 and P1-0112 programs and N7-077 project), and a DFG Postdoctoral Fellowship to SF (FI 2152/1-1).

## Conflict of Interest

The authors declare that the research was conducted in the absence of any commercial or financial relationships that could be construed as a potential conflict of interest.
