# Supplementary material for: Early Responses of Brassica oleracea Roots to Zinc Supply Under Sufficient and Sub-Optimal Phosphorus Supply
Source: Front Plant Sci. 2020 Jan 9;10:1645. doi: 10.3389/fpls.2019.01645 (PMC6962232; doi:10.3389/fpls.2019.01645)
Supplement: Supplementary file 1 [file DataSheet_1.docx]

**Supplementary material to:**

**Early responses of *Brassica oleracea* roots to zinc supply under sufficient and sub-optimal phosphorus supply**

**Paula Pongrac^1,2,*,¥^, Sina Fischer^3,¥^, Jacqueline A. Thompson^1^, Gladys Wright^1^, Philip J. White^1,4^**

^1^Ecological Science Group, The James Hutton Institute, Invergowrie, Dundee DD2 5DA, UK

^2^Jožef Stefan Institute, Jamova 39, SI-1000 Ljubljana, Slovenia

^3^Future Food Beacon of Excellence and School of Biosciences, University of Nottingham, Sutton Bonington Campus, Loughborough, LE12 5RD, UK

^4^Distinguished Scientist Fellowship Program, King Saud University, Riyadh 11451, Saudi Arabia

*Corresponding author:

Jožef Stefan Institute, Jamova 39, SI-1000 Ljubljana, Slovenia

Tel: +386-51-222-963; Fax: +386 477 31 51

Email: [paula.pongrac@](mailto:paula.pongrac@)ijs.si

¥ these authors contributed equally to the work


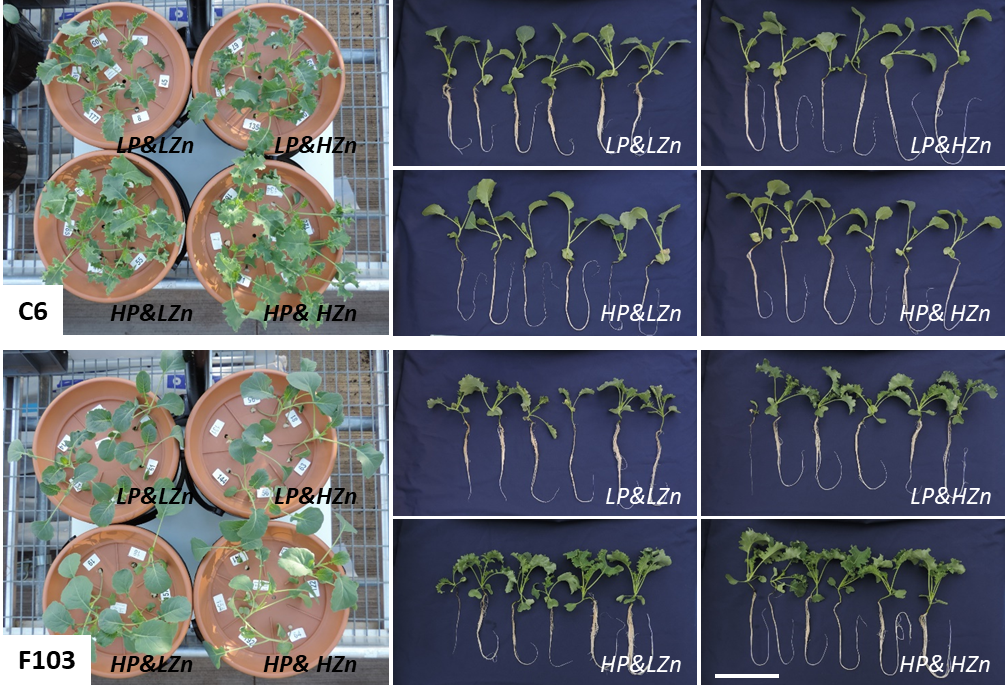


**Figure S1** Growth of *Brassica oleracea* accessions C6 and F103 in hydroponics for two weeks with contrasting combinations of phosphorus (P; Low P (LP) and High P (HP)) and zinc (Zn; Low Zn (LZn) and High Zn (HZn)) supply used for the quantification of plant ionome and root gene expression analysis. Scale bar indicates 10 cm


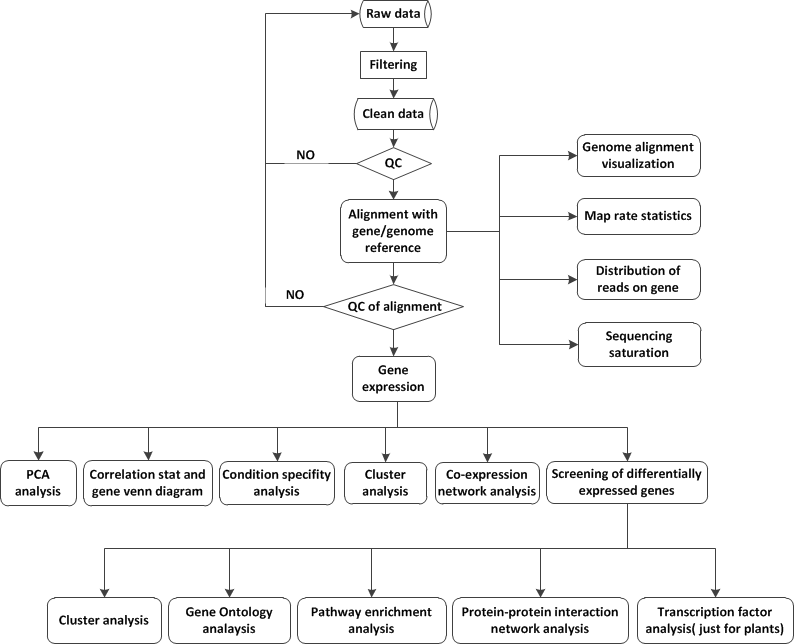


**Figure S2** Schematic representation of BGI Bioinformatics analysis pipeline


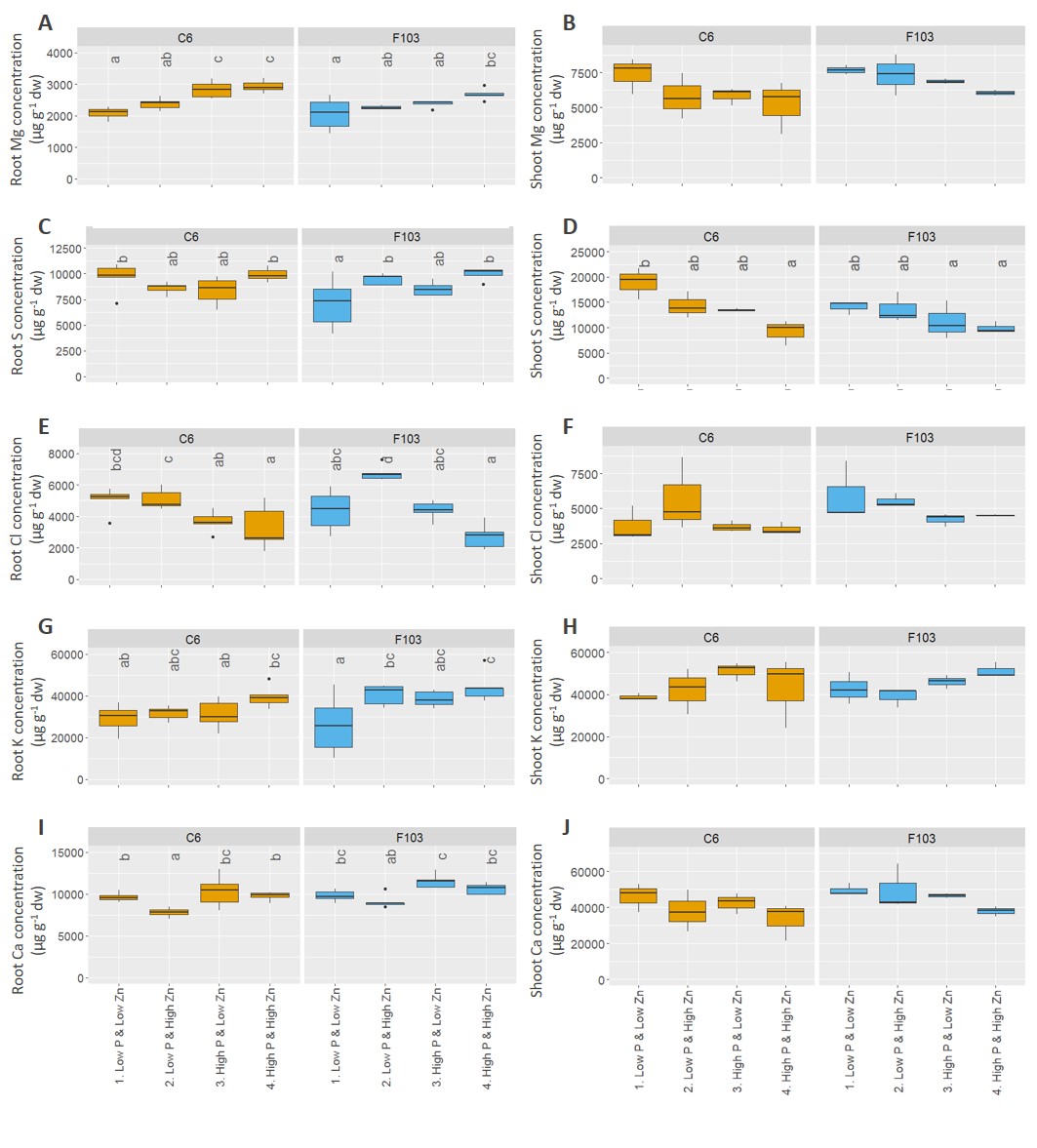


**Figure S3 Effect of contrasting combinations of P and Zn supply on two *Brassica oleracea* accessions.** Plants were grown hydroponically for two weeks after which Mg (**A, B**), S (**C, D**), Cl (**E, F**), K (**G, H**) and Ca (**I, J**) concentrations were determined in roots (**A, C, E, G, I**) and shoots (**B, D, F, H, J**). Shown are Tukey boxplots representing 25th and 75th percentile of the data with the centreline representing the median and whiskers representing the 5th and 95th percentile (for each accession and each treatment n=5-9 for root simples and n=3 for shoot samples). Significant differences between accessions and treatments are indicated by different letters above the box-plots (Two-way analysis of variance followed by Tukey post-hoc test at p<0.05). dw, dry weight


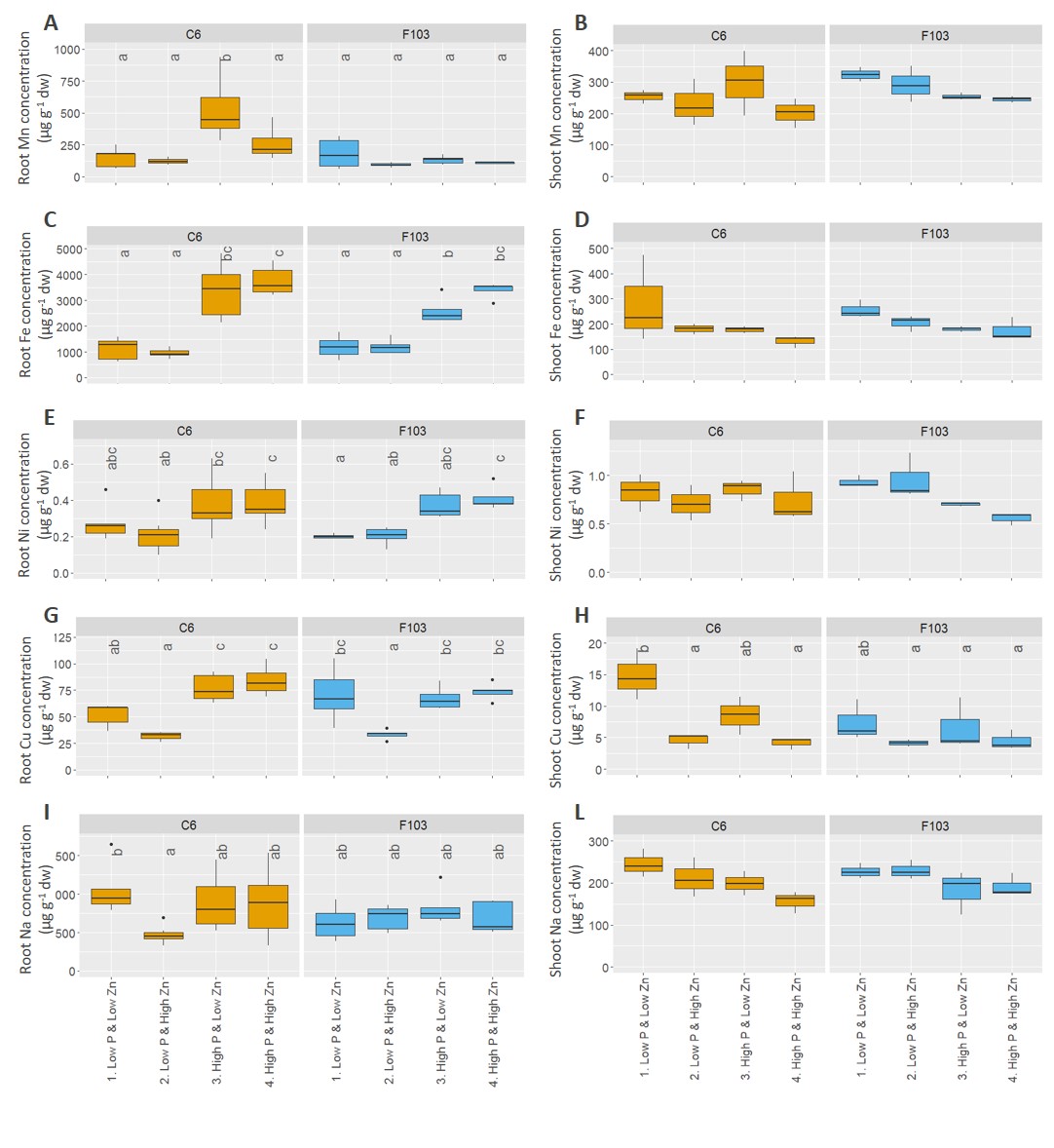
**Figure S4 Effect of contrasting combinations of P and Zn supply on two *Brassica oleracea* accessions.** Plants were grown hydroponically for two weeks after which Mn (**A, B**), Fe (**C, D**), Ni (**E, F**), Cu (**G, H**) and Na (**I, J**) concentrations were determined in roots (**A, C, E, G, I**) and shoots (**B, D, F, H, J**). Shown are Tukey boxplots representing 25th and 75th percentile of the data with the centreline representing the median and whiskers representing the 5th and 95th percentile (for each accession and each treatment n=5-9 for root simples and n=3 for shoot samples). Significant differences between accessions and treatments are indicated by different letters above the box-plots (Two-way analysis of variance followed by Tukey post-hoc test at p<0.05). dw, dry weight


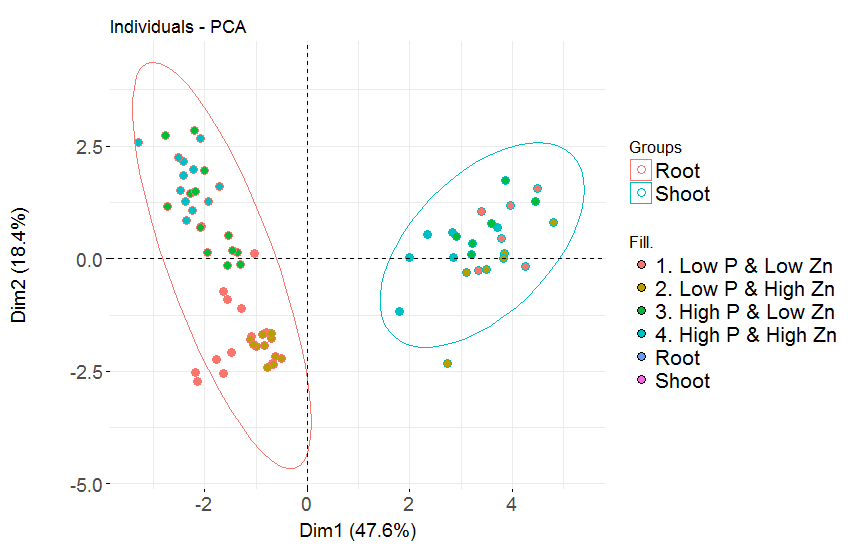


**Figure S5 Principal component analysis (PCA) of the root and shoot ionomes of two *Brassica oleracea* accessions (C6 and F103).** The dry weight and concentrations of Na, Mg, P, S, Cl, K, Ca, Mn, Fe, Cu, Zn and Ni in roots and shoots of the C6 and F103 accessions grown hydroponically for two weeks with contrasting combinations of P and Zn supply were included in the PCA to detect major effects on the variations observed. The PCA plot depicts the first two PCs (Dim1 and Dim2) for response variables grouped by plant organ (root and shoot). The colour of the ellipse edge indicates the group: pink for root and blue for shoot. For shoots n=3 and for roots n=4-9 for each accession and each treatment)
